# Fabrication of well-defined magnetic microporous polymeric monoliths using simple non-aqueous emulsification technique

**DOI:** 10.1038/s41598-025-90345-0

**Published:** 2025-03-03

**Authors:** Aya A. Karrar, Fouad Taha, Hisham A. Essawy, Amro K. F. Dyab, Ahmed I. A. Abd El-Mageed

**Affiliations:** 1 Colloids and Advanced Materials Group, Chemistry Department, Faculty of Science, Minia University, Minia, 61519 Egypt; 2 Department of Polymers and Pigments, National Research Centre, Dokki, Cairo, 12311 Egypt; 3 Department of Chemistry, Nazarbayev University, Kabanbay Batyr 53, Astana, 010000 Kazakhstan; 4 Chemistry Department, Faculty of Science, Galala University, Galala City, 43511 Suez Egypt

**Keywords:** Magnetic polymer, Monolith, Microporous, Oil-in-oil emulsion, Magnetite, Chemistry, Materials science, Nanoscience and technology

## Abstract

The current work describes a novel route for preparation of robust polymeric monolithic structures exhibiting magnetic properties via emulsification of a polar glycerin oil in a polymerizable hydrophobic oil of styrene as oil/oil (o/o) emulsion technique. Hydrophilic magnetite nanoparticles were first prepared via the co-precipitation method and then converted to organophilic using oleic acid as a surface coating material. The FT-IR provided evidence on the covering of the particle’s surface and also revealed some hydrophilic OH groups co-exist, implying a probable amphiphilic character is acquired. The organophilic particles act efficiently as Pickering stabilizers for glycerin/styrene emulsion systems. Styrene, a polymerizable oil, could be subsequently polymerized at 70 °C in the presence of an oil-soluble thermal initiator such as 1,1-azobiscyclohexanecarbonitrile (vazo). Scanning electron microscopy (SEM) confirmed the formation of well-defined, highly porous polymeric monoliths, in which the distribution of the pores within the monolith further supported that they were prepared via well-emulsified glycerin drops in the styrene phase as a precursor. Additionally, the EDX revealed the presence of the iron element distributed evenly within the monolith. The thermogravimetric analysis (TGA) revealed a slight resistance to thermal degradation over a narrow range up to 150 °C with respect to pure polystyrene, whereas beyond this temperature the degradation behavior proceeded almost typically as for pure polystyrene. The ferromagnetic resonance spectroscopy (FMR) indicated the acquisition of the magnetic property by the produced monolith structure. For the best of our knowledge, it is the first article of its type investigating the fabrication of polymeric monolithic structures from non-aqueous emulsions.

## Introduction

Crosslinked porous polymeric materials are particularly worthwhile in wide- ranging fields^1–3^. Such materials acquired an enduring porous structure that developed over the synthetic steps and survived while in the dry state. The inner macroporosity is distinguished by interconnected network, which allows permeation through the matrix. These porous structures are frequently prepared in the form of well-defined even beads via suspension/dispersion polymerization^4,5^. Nevertheless, a limitation of this route is the shortening of its application to chromatographic separations of large molecules.

On the other side, a monolith is defined as a continuous object whose morphological features and porous structure can be altered in a wide range^6^. It is generally prepared by polymerization of monomers with inorganic or organic based skeleton^6,7^, which presents an alternative promising technique for developing continuous, porous polymeric structures^7,8^.

The produced monolithic structures were employed in a broad diversity of applications like high-performance liquid chromatography (HPLC)^9^, solid phase extraction^7,9^, high-performance membrane chromatography (HPMC)^7^, capillary electro-chromatography^7,9^, molecular imprinting^9,10^, gas phase catalytic reactions^11^, catalytic activity with high reusability^12,13^, efficient dye removal from waste water^14^ as well as high-throughput bioreactors^15,16^. The ease of regeneration constitutes an interesting feature for these types of materials^17^.

The majority of synthetic routes until now encompassed free radical polymerization of methacrylates or styrene based cross-linkers (e.g., ethylene glycol dimethacrylate, divinyl benzene)^4^. Careful selection of solvent mixtures, either solvating or non-solvating allows realistic regulation of the porosity of the produced monoliths. Some tailored recipes can procure a distribution of small, diffusive pores (< 100 nm), interconnected with larger, flow-through pores with diameters covering 700–2000 nm^18^. A distinct feature of these processes is that they allow straight attainment of the anticipated porous structures, comprising both wide pores chromatography columns and narrow pores capillaries at the same time.

Oil- in water (o/w) and oil- in oil (o/o) emulsions are versatile techniques for many reactions because they are inexpensive, non-toxic, and consistent^19–22^. Our research focuses on non-aqueous systems, in which a polar solvent such as glycerin substitutes the water. These systems offer viable alternatives to aqueous solutions in scenarios where water presence is undesirable [11]. While instances of non-aqueous emulsions, or oil-in-oil emulsions, are relatively uncommon, they present significant advantages in applications where the water can be detrimental, such as the biomedical and cosmetic fields [12].

The current work presents a novel simple, cost-effective route for preparation of microporous monoliths via oil (glycerin) in oil (styrene monomer) emulsion followed by free radical polymerization of the continuous monomer phase (styrene) in presence of organophilized magnetic nanoparticles (iron oxide). The developed route is expected to provide strength and wide varieties of well-tailored continuous microporous materials. The magnetite nanoparticles and corresponding polymeric monoliths were characterized using many analytical techniques, including fourier transform infrared spectroscopy (FTIR), thermo-gravimetric analysis (TGA), scanning electron microscopy equipped with energy dispersive X-ray spectroscopy (SEM-EDX), X-ray diffraction (XRD), and ferromagnetic resonance spectroscopy (FMR).

## Materials and methods

### Materials

Styrene with a purity of 99% was obtained from Merch KGaA, Germany, and purified before use to remove the stabilizer using an aqueous solution of sodium hydroxide. Anhydrous ferric chloride (98% purity) was procured from Alpha Chemika, India. Ferrous sulphate heptahydrate (99% purity) and sodium hydroxide were provided from Fine Chemicals, Al-Gomhoria company, Egypt. High-purity oleic acid (≥ 99%) was supplied by Sigma-Aldrich Labor chemikalien GmbH. Ammonia solution (33%), and glycerin (98%) were obtained from ADWIC, El Nasr Pharmaceutical Chemicals Co., Egypt. Ethyl alcohol with a purity of 96% was obtained from PIOCHEM, Egypt. Potassium hydroxide (85% purity) was purchased from Chem. Lab NV, Belgium. Commercial-grade 1,1-azobiscyclohexanecarbonitrile (vazo), used as a polymerization initiator, was supplied by Santa Cruz Biotechnology, Inc., Germany.

### Methods

#### Preparation of organophilized magnetite nanoparticles (OCMNs)

A co-precipitation method was used to synthesize organophilized magnetite nanoparticles. Iron(III) chloride (3.25 g) and iron(II) sulphate heptahydrate (2.78 g) were dissolved in deionized water (40 mL) and reacted with ammonium hydroxide solution (12 mL, 33% w/w), to yield hydrophilic magnetite nanoparticles (Fe_3_O_4_)^23^. Subsequently, these nanoparticles were converted into a hydrophobic form by treating them with oleic acid according to a previously established protocol^24^. The reaction was maintained at 70 °C for a total of 120 min, with an increased stirring rate during the latter hour. Following cooling to room temperature, the magnetite particles were isolated using magnetic decantation provided by a neodymium magnet. The resulting black precipitate was rigorously washed with water and ethanol before being dried in an oven at 60 °C. The produced organophilic nanoparticles will be termed as OCMNs throughout the next sections in this work.

#### Fabrication of magnetic microporous polymeric monoliths

The oil-in-oil emulsions were formed by dispersing a pre-determined amount of OCMNs and vazo in 8 mL styrene (the continuous phase), then 2 mL glycerin (the dispersed phase) were dispersed within the previous mixture under constant stirring by employing Wise Tis HG15D and Ivymen CY-500 ultrasonic homogenizers (8000 rpm for 3 minuities) to achieve the emulsification. The emulsion stability through time was studied over a month. However, All the samples had almost the same height (immediately after preparation) but through time, the emulsion height changed as the stability changed. The drop-in stability is followed in the form of creaming height (calculated by recording the emulsion height divided by total height of sample × 100). It’s worth to mention that the pH was crucial to achieve a stable emulsification, where at pH = 11 of the glycerin based aqueous phase a stable emulsion could be formed. The concentration of OCMNs varied between 1 and 5 wt% while maintaining a constant vazo concentration of 2 wt%, based on the content of styrene continuous phase. The polymerization was initiated while employing a mechanical stirrer (Ingenieurbüro CAT M-Zipperer GmbH) by heating the emulsion to 70 °C for 20 h to ensure completion of the polymerization, to yield eventually solid monoliths.

### Characterizations

The extent of organophilicity of the magnetite particles after treatment with oleic acid was predicted by undergoing contact angle measurements between a water drop on compressed discs of the particles with a diameter of 12.56 mm and a thickness of 2.93 mm using magnification lens with image-J software. Optical microscopy utilizing an OPTIKA B-293 microscope and Optikam B5: 4083 digital camera provided visual characterization of the emulsion samples. In addition, water drop method was undertaken to ensure the type of formed emulsion. Fourier transform infrared (FTIR) spectroscopic analysis was performed on a Thermoscientific NICOLET IS 10 spectrometer within a wavenumber range of 400–4000 cm⁻¹ using potassium bromide as a background material. Scanning electron microscopy (SEM), using JEOL model (JSM-5400), equipped with gold sputtering capabilities, was utilized to examine the surface morphological features of the samples at different magnifications. The elemental composition of the samples was determined via energy dispersive x-ray spectroscopy (EDX) integrated with the JEOL JSM-IT200 microscope. The Thermodegradation behavior was examined through thermogravimetric analysis (TGA) using a Shimadzu TGA-50 H instrument under nitrogen flow at a controlled heating rate of 10 °C/min. X-ray diffraction (XRD) analysis with Cu Kα radiation source was conducted on a Philips pw1710 diffractometer for investigating the crystalline structure and determination of the particles size by applying Debye-Scherrer Eq. (d = Kλ/β cos θ). The magnetic behavior of the samples was assessed at room temperature using a Lake Shore Model 7410 vibrating sample magnetometer (VSM) with applied magnetic fields of up to 20 kOe.

## Results and discussion

### Preparation of organophilized magnetite nanoparticles (OCMNs)

**Fig. 1:**
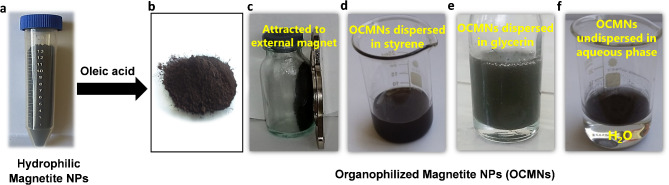
(**a**) Hydrophilic magnetite nanoparticles prepared by the co-precipitation method, (**b**) OCMNs as prepared, (**c**) OCMNs show a magnetic response to an external magnet, (**d**,**e**) The OCMNs are well-dispersed in the oil phases (styrene and glycerin, respectively), (**f**) OCMNs are poorly dispersed in the aqueous medium.

The target magnetic nanoparticles were prepared using co-precipitation method, where iron(III) and iron(II) salts were mixed together in the presence of ammonium hydroxide solution as a basic medium, then the resultant hydrophilic magnetite nanoparticles (Fig. 1a) were then treated using oleic acid to yield OCMNs, as depicted in Fig. 1b. It is noteworthy to observe that the magnetic identity of the particles was not influenced by the coating with oleic acid (Fig. 1c) whereas their dispersibility in non-polar phases was enhanced following the surface organophilization (Fig. 1d, e) on the contrary to polar media (Fig. 1f).

**Fig. 2:**
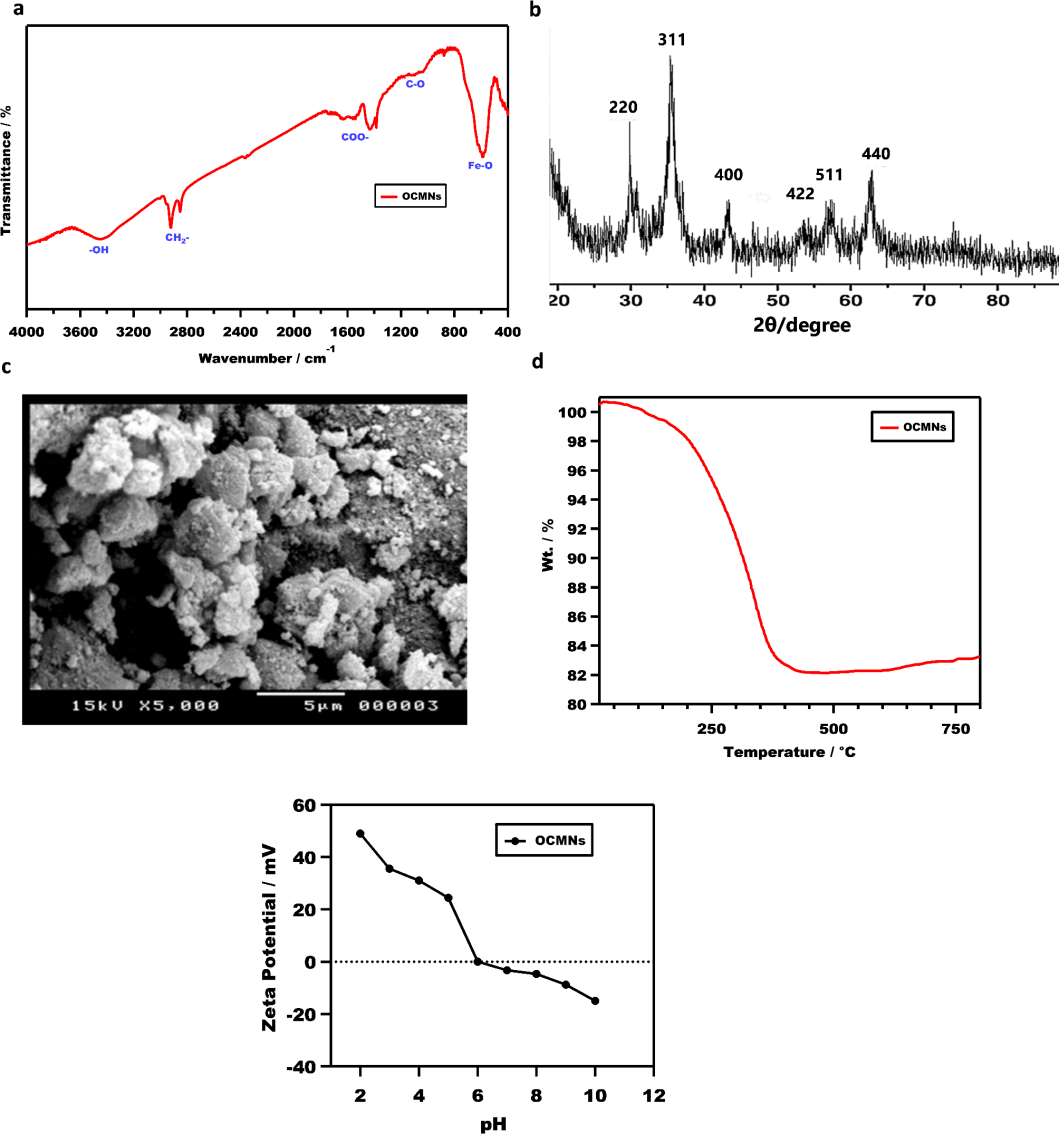
(**a**) FTIR spectrum, (**b**) X-ray diffraction (XRD) pattern, (**c**) SEM micrograph, (**d**) TGA traces, for the OCMNs. (**e**) Dependence of zeta potential on the pH of OCMNs (the IEP was determined at pH = 6.3).

The FTIR spectrum of the magnetite nanoparticles after treatment with oleic acid is presented in Fig. 2a. The Fe-O band appeared at 591 cm^−1^, which is characteristic to Fe_3_O_4_. The asymmetric and symmetric CH_2_ stretching band appeared at 2922 cm^−1^ and 2852 cm^−1^, respectively, representing a slight shift with respect to the original position in case of pure oleic acid (2925 cm^−1^ and 2856 cm^−1^)^25^. Two bands can be recognized at 1570 cm^−1^ and 1662 cm^−1^, which are characteristic of C=O asymmetric and symmetric stretching. Their shift from the original position in case of pure oleic acid at 1714 cm^−1^^25^ may indicate the involvement in some interactions for achieving the adsorption on the surface. Furthermore, the appearance of C-O a little bit stretched at 1125 cm^−1^ can support this postulation^24^. This can additionally reveal the presence of oleic acid carboxylate groups on the surface boosting the chemisorption. The wide band representing OH groups at 3450 cm^−1^ confirms the surface of magnetite nanoparticles is still maintaining some hydrophilic groups, which allows it to play the role of surfactant due to its amphiphilic nature.

The modified sample was also investigated using XRD as shown in Fig. 2b, which exhibit sharp typical peaks of the magnetite nanoparticles (at 2θ = 30.15, 35.46, 43.09, 53.55, 56.97 and 62.67). This explains that the crystalline identity of the particles was maintained following the treatment. The OCMNs particle size calculated by applying Debye-Scherrer equation was 10.46 nm.

The SEM micrographs taken for the OCMNs in comparison with their state before treatment^26,27^ show formation of clusters and aggregations on the surface, and increased level of roughness, Fig. 2c. This signifies the coverage of the particles surface and emergence of hydrophobic-hydrophobic interactions due to the presence of adsorbed oleic acid molecules on the surface to form the clusters network leading to development of surface roughness.

The modified samples were also examined using TGA for their decomposition behavior after the surface coverage by an organic layer of oleic acid (Fig. 2d). The degradation profile exhibited a systematic loss starting from 50 °C up to 400 °C, then it reached a plateau in which the total attained weight loss was around 16%. The residual weight (84%) is representing the inorganic magnetite component, indicating the removed weight represent the adsorbed oleic acid layer(s).

The isoelectric point (IEP) of OCMNs, defined as the pH at which the net surface charge is zero, was determined using zeta potential measurements. As illustrated in Fig. 2e, the IEP of OCMNs was found to be 6.3, consistent with previously published findings^24,28–30^. This indicates that when the pH is below the IEP, the magnetite nanoparticles exhibit a positive surface charge, whereas at pH values above the IEP, they acquire a negative surface charge.

**Fig. 3:**
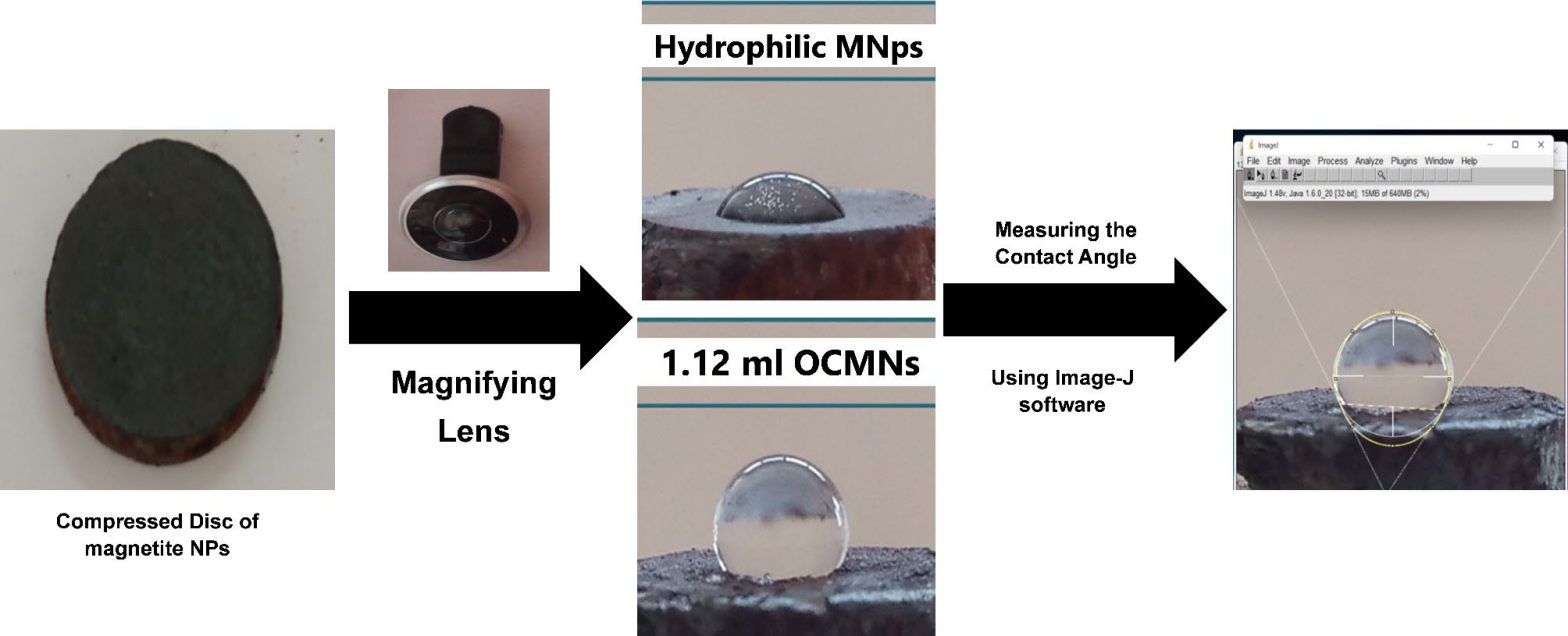
Contact angle measurements for the prepared magnetite nanoparticles before and after the treatment with oleic acid.

To further elucidate the acquired hydrophobicity of the particles after treatment, the contact angle measurements were conducted between water drops on the surface of discs prepared by compression of the particles before and after treatment (Fig. 3), where it was found that it increased after the treatment to 118.8°, compared with 59.7° before the treatment, indicating the potential of using the particles now to act as surface active elements due to their gained amphiphilic character.

### Preparation of glycerin-in-styrene emulsion stabilized with OCMNs

Oil/oil (o/o) emulsions are formed between two immiscible non-polar/polar aprotic organic solvents. A carefully designed amphiphilic block is mostly needed as surfactant to stabilize these types of emulsions^19,31–34^. These non-aqueous emulsions allow the use of water-sensitive additives and ensure limitations of side products usually encountered in aqueous media^22^. In The current work, the amphiphilic magnetite nanoparticles were encouraging for use to stabilize glycerin in styrene emulsion, considering their well dispersion in styrene and much less dispersibility in glycerin to act as Pickering stabilizers^8,23,35–37^.

Several trials were attempted for formation of stable emulsions using different levels of the organophilic nanoparticles but all failed unless the pH of the glycerin based aqueous phase was adjusted either above or below the isoelectric point of the nanoparticles, which was determined around 6.3 (Fig. 2e) that in agreement with many previous reports^24,28–30^.

**Fig. 4:**
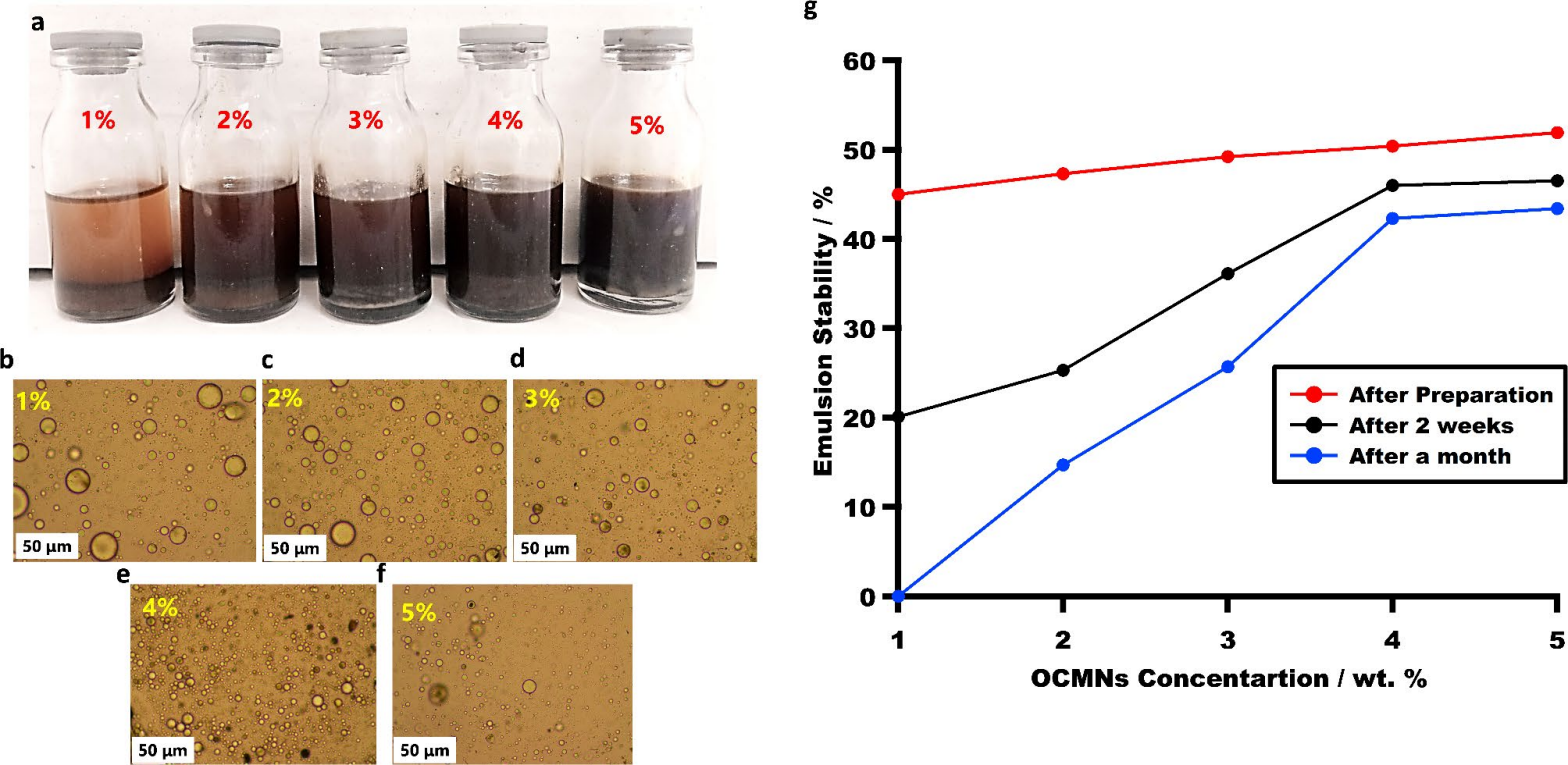
(**a**) Digital photo for the glycerin-in-styrene emulsion samples stabilized with OCMNs (1–5 wt%). (**b**–**f**) Optical images for the prepared glycerin-in-styrene emulsion stabilized with OCMNs, where (**b**) 1 wt%, (**c**) 2 wt%, (**d**) 3 wt%, (**e**) 4 wt% and (**f**) 5 wt%. (**g**) The emulsion stability vs. Time.

At pH = 4 a stable emulsion could be successfully attained but the polymerization failed. Eventually, at pH = 11, a stable emulsion could be formed as can be shown in Fig. 4a for all employed loadings of the particles as stabilizer (1–5 wt%). The drop test confirms that styrene is the continuous phase in all the prepared emulsion samples.

Figure 4b–f show the optical images of the glycerin-in-styrene emulsion stabilized with OCMNs 1, 2, 3, 4, 5 wt%, in which the average emulsion droplet size was around 30, 22, 15, 10, 8 μm, respectively. It can be noticed that the droplet size of the glycerin phase became more well dispersed and acquired more uniform smaller size for a loading of 5 wt% of the particles based on the styrene phase. The collapse of the droplets size with more loading of the particles corroborates their role as dispersing fillers due to their surface-active characters.

Since the fabrication of monolith from non-aqueous emulsion system is a bit novel, it was important to prepare a stable O/O emulsion precursor, avoiding the emulsion instability phenomena (i.e. creaming, sedimentation as well as phase separation). Therefore, the emulsion stability versus time was studied over one month after preparation (Fig. 4g). The droplet stability is followed in the form of creaming height (calculated by recording the emulsion height divided by total height of sample × 100). Immediately after preparation the prepared emulsion samples had almost the same creaming height, however, through the time the sample stabilized with 1% OCMNs completely separated after 1 month whereas the stability of the emulsion stabilized with 5% OCMNs reached 43.4% (as shown in Fig. 4g). This indicates that the emulsion stability changes with time, but it can be controlled and/or enhanced by increasing the OCMNs concentrations. This means that there is plenty of time available to prepare the desired monolith from its stable emulsion precursor. In line with that, the bare magnetite nanoparticles are hydrophilic as revealed from the contact angle measurements and did not form stable suspension in oils. Nevertheless, the wettability of the magnetite particles was optimized via surface modification resulting in hydrophobic particles that were able to stabilize the glycerin-styrene interface.

### Fabrication of magnetic microporous polymeric monoliths

Interestingly, the increase in the temperature in this case resulted in successful polymerization of the styrene continuous phase, which seems to be proceeding favourably at pH values above 6.3. It is thought that the polymerization process could not be proceeded at pH = 4 due to the extent of electrostatic repulsion on the particles surface was very high, causing disturbance in the system during the polymerization, therefore not to allow the monomer droplets to approach each other in such a way to polymerize, while at pH much above the isoelectric point (pH = 11) the extent of electrostatic repulsion between the droplets surfaces carrying negative charges was not that high to hinder the polymerization.

By the end of the polymerization process of the continuous styrene phase, the glycerin phase would be expelled out due its non-polymerizability, leaving behind microporous structure distributed evenly within the produced monolithic polymeric system whereas the functionalized organophilic particles would remain well-dispersed within this phase as reinforcing fillers, in which their inherent magnetism will tentatively be conveyed to the produced polymeric monolith.

**Fig. 5:**
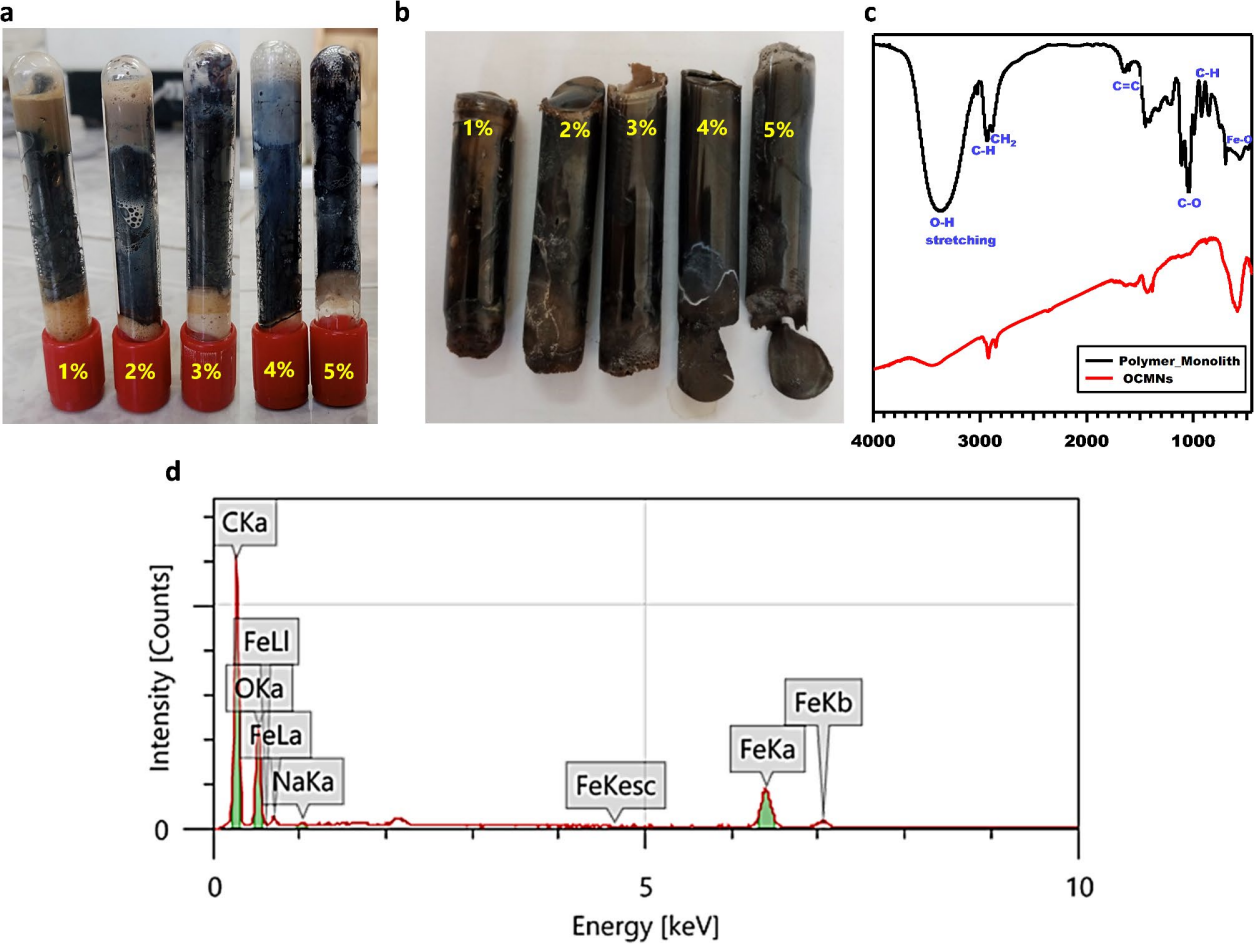
(**a**,**b**) Digital photos for the glycerin-in-styrene derived polymeric monoliths stabilized with 1–5 wt% OCMNs. (**c**) FTIR spectra of glycerin-in-styrene derived polymeric monolith stabilized with 5 wt% OCMNs, compared to OCMNs. (**d**) EDX Analysis for glycerin-in-styrene derived polymeric monolith stabilized with 5 wt% OCMNs.

A black, opaque solid was separated after 20 h from start of the polymerization process (Fig. 5a,b), indicating the polymerization was almost completed. The FTIR spectra of the polymeric monoliths (compared to OCMNs) is presented in Fig. 5c. The Fe-O band was shifted from 591 to 566.58 cm^−1^ which is characteristic for Fe_3_O_4_. The asymmetric and symmetric CH_2_ vibrations were shifted from 2922, 2852 to 2933, 2884 cm^−1^, respectively. The C-O single bond stretching was shifted from 1125 to 1111 cm^−1^. Two absorption peaks at 3059 and 3025 cm^− 1^ attributed to aromatic C-H stretching vibration absorption. Moreover, three peaks were identified for the aromatic C = C stretching vibrations at 1600, 1492, and 1452 cm^− 1^, indicating the presence of benzene rings, which proves the formation of polystyrene. The absorption peaks at 754 and 697 cm^− 1^ corresponding to C-H out-of-plane bending explain that there is only one substituent in the benzene ring. In addition, the absorption peak at 3382 cm^−1^ illustrates the existence of hydroxyl groups, which appeared broader than usual. Figure 5d shows the EDX analysis of the polymeric monolith stabilized originally with 5 wt% OCMNs, where the peaks of carbon, oxygen and iron are clearly present, indicating the successful fabrication of polystyrene/OCMNs composite. From the EDX, the atomic percentages of C, O, Fe are of 64.6, 33.6, 1.6%, respectively.

**Fig. 6:**
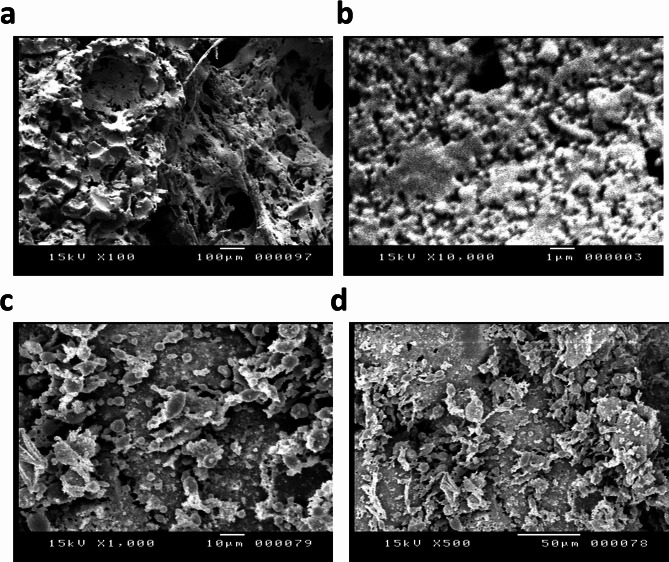
SEM images for glycerin-in-styrene polymeric monolith stabilized with OCMNs, (**a**,**b**) 1 wt%, (**c**,**d**) 5 wt%.

Figure 6a–d shows the SEM micrographs of the obtained polymeric monoliths. The high observed porosity indicates the removed emulsified glycerin drops following the completed polymerization acted efficiently as porogen material (Fig. 6a,b). It is obvious that the material appeared sufficiently robust, lacking of distinct cracks. The almost mono-disperse size of the pores reveals the emulsion precursor was remaining stable over the polymerization interval and did not encounter droplets flocculation. It can be recognized that the organophilic particles concentration has a strong influence on the porous structure of the formed monoliths, where at high loading of the particles (5%), the porosity of the monoliths declined dramatically and seemed to be blocked by the particles (Fig. 6c,d). In addition, it is known that for Picketing emulsions the size of the formed droplets is inversely proportional to the concentration of stabilizing solid particles.

**Fig. 7:**
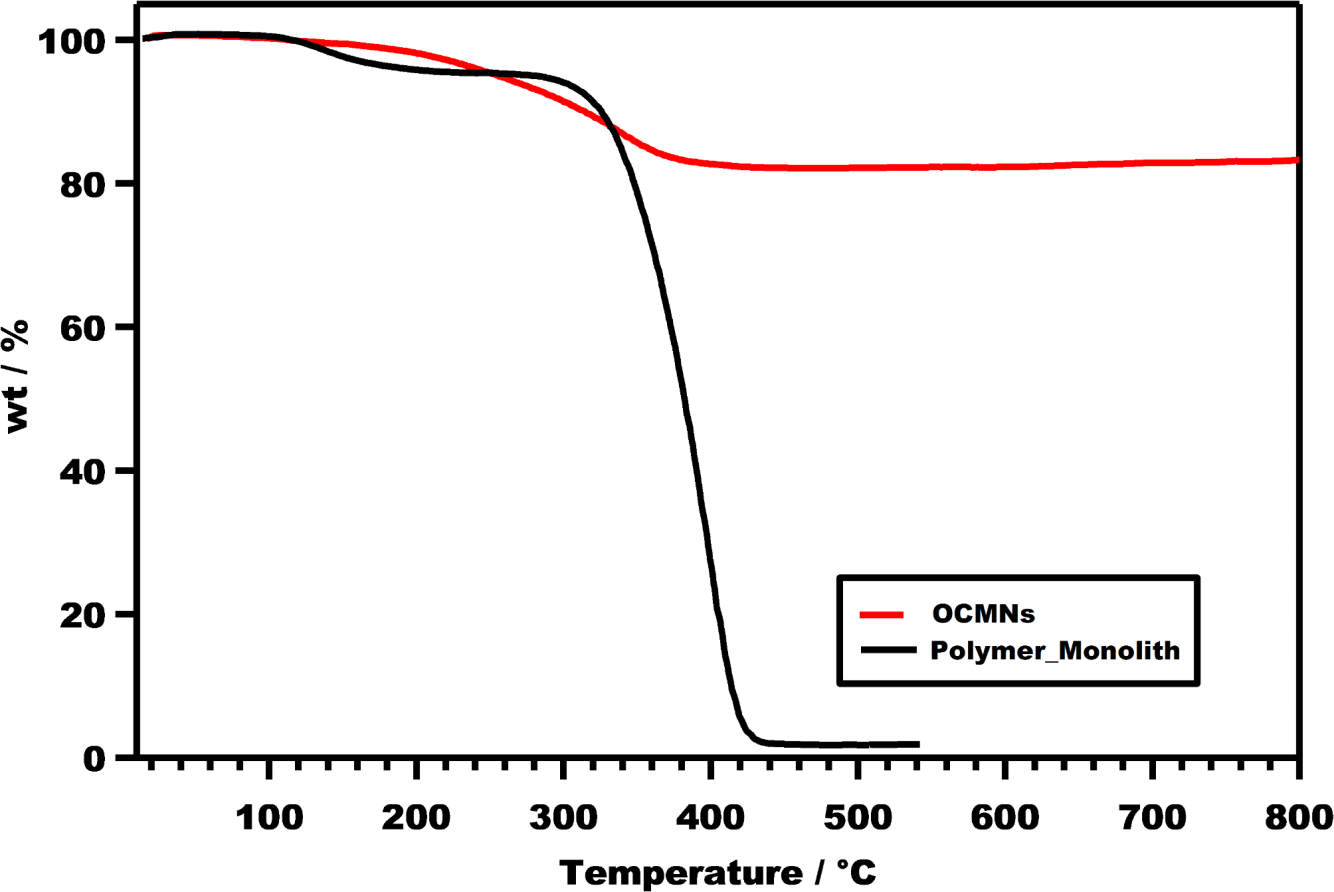
Thermal degradation of OCMNs as well as the polymeric monolith stabilized with OCMNs.

The polymeric monolith samples were also examined using TGA for their decomposition in comparison with the OCMNs (Fig. 7). The degradation behavior of the formed monolith was almost very similar to that of OCMNs in the range 50 to 344 °C, with a noticeable slight faster degradation accounting for 6% total loss in this stage. The degradation proceeded much rashly and faster beyond 344 °C, in which the attained weight loss up to 430 °C reached additional 91 wt %, leaving behind a residual weight accounting for 3%, which represents mostly the carbonized inorganic phase of the magnetite. This content can be compared with the employed ratio of OMCNs used for emulsion stabilization, where it can be realized that almost all the stabilising OCMNs were incorporated effectively within the channels and cells of the monolithic polymer formed without encountering noticeable loss.

**Fig. 8:**
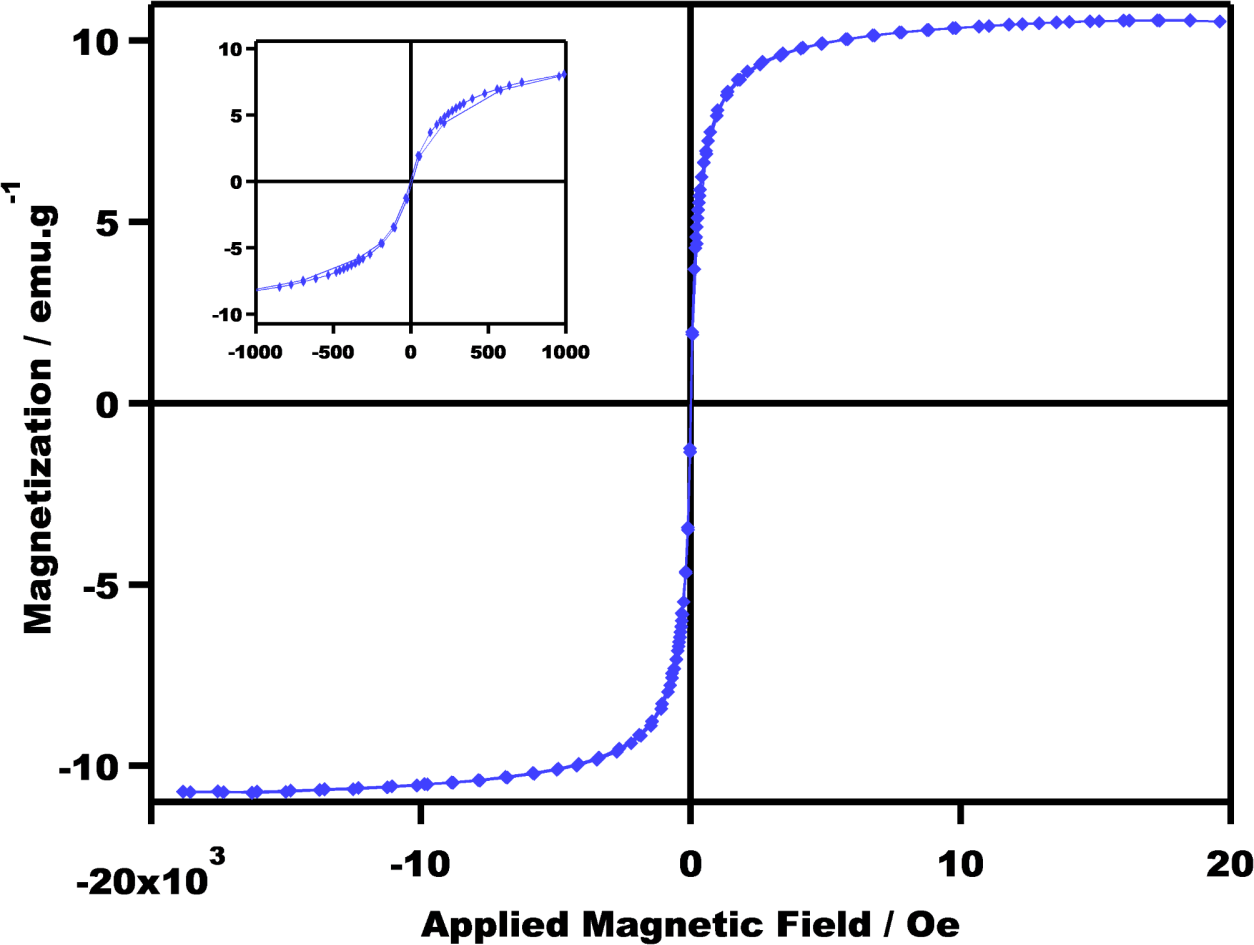
The magnetization curve of a polymeric monolith containing 5 wt% OCMNs was investigated at room temperature (298 K). A magnetic field strength ranging from ± 20 kOe was applied. The resulting hysteresis loop, which demonstrates the material’s magnetic behavior, is clearly depicted in an enlarged inset focused on the region around zero magnetic field.

Figure 8 presents the magnetization curve for a polymer monolith measured at room temperature (298 K) under an applied magnetic field ranging from ± 20 kOe. The observed butterfly-shaped hysteresis loop confirms the ferromagnetic behavior of the material. This indicates that the polymer exhibits micromagnetic properties. Table 1 summarizes key magnetic parameters extracted from the curve, including saturation magnetization (*M*_*s*_), remanent magnetization (*M*_*r*_), and coercivity (*H*_*c*_). The wider hysteresis loop observed in the composite compared to pure OCMNs as reported in the literature^24,28^, suggests that the distribution of organophilic magnetite within the polystyrene matrix, coupled with interaction forces between particles, contributes to this phenomenon.

**Table 1:** Magnetic parameters were obtained at 25 °C for the polymer monolith with 5% OCMNs as a stabilizer.

Parameter	Value
Area-total	1227 erg/g
Coercivity (*H*_*c*_)	5.8834 G
Flatness	1.3244
*H*_*c*_, Negative	− 2.4897 G
*H*_*c*_, Positive	9.2772 G
Magnetization (*M*_*s*_)	10.653 emu/g
*M*_*r*_, Negative	− 0.37486 emu/g
*M*_*r*_, Positive	9.96E−02 emu/g
M_s_, Negative	− 10.743 emu/g
M_s_, Positive	10.563 emu/g
Retentivity (*M*_*r*_)	0.23725 emu/g
Squareness	2.23E−02

Eventually, it can be recommended that using a volume fraction of 2% of glycerin/styrene and 5 wt% of OCMNs as a stabilizing filler can be considered as an optimized conditions in terms of emulsion stability (before polymerization) and handling stability, porosity, thermal stability as well as magnetization for the resulting polymerized monoliths. We demonstrated a proof of concept in the current study and will further explore these optimization conditions in the future study.

## Conclusion

Emulsification of polar oils such as glycerin in a polymerizable hydrophobic oil like styrene can be successfully achieved using a Pickering stabilizer system incorporating organophilic magnetite nanoparticles. The dual hydrophilic/hydrophobic character of the particles enabled them to achieve good stabilization of this emulsion precursor. Subsequent polymerization of the polymerizable continuous phase allows formation of a polymeric monolith material exhibiting well-defined porosity, resulting from the migration of the glycerin drops during the successive development of polymerization. The homogeneous distribution of the organophilic nanoparticles allows also the produced polymeric monolith to effectively acquire the reinforcing action provided by the particles in addition to a stable magnetic response inherited due to their inherent magnetism. The prepared polymeric monolith can be used in many potential applications such as in separation science, hyphenated techniques as well as in bio-catalysis.

## Data Availability

Data is provided within the manuscript.
